# Long-term intra- and inter-individual biological variation of serum lipid of HIV-infected and uninfected men participating in the Los Angeles Multi-Center AIDS Cohort Study (MACS)

**DOI:** 10.1186/s12944-022-01668-0

**Published:** 2022-07-27

**Authors:** Najib Aziz, David W. Gjertson, Matthew J. Mimiaga, Chantel D. Azarkman, Rey Soto, Nicole Alexopoulos, Roger Detels

**Affiliations:** 1 Department of Epidemiology, UCLA, Fielding School of Public Health, Los Angeles, CA 90095-1772 USA; 2 Department of Biostatistics, UCLA, Fielding School of Public Health, Los Angeles, CA USA; 3 Department of Psychiatry & Biobehavioral Sciences, UCLA, David Geffen School of Medicine, Los Angeles, CA USA; 4 Department of Medicine, UCLA, David Geffen School of Medicine, Los Angeles, CA USA

**Keywords:** Biological variation, Cholesterol, CV_I_, CV_G_, HDL-C, Triglycerides

## Abstract

**Background:**

To assess the long-term biological coefficient of variation within individuals (CV_I_) and between individuals (CV_G_), effect of aging and cholesterol lowering drugs on blood levels of lipids in HIV-1-infected and -uninfected men.

**Methods:**

Bloods were analyzed every six months over 17 years for total cholesterol (TC), triglycerides (TGs), high-density lipoprotein cholesterol (HDL-C) and low-density lipoprotein cholesterol (LDL-C) in 140 HIV-uninfected (38–66 years old) and 90 HIV-treated infected (48–64 years old) white Caucasian men to examine CV_I_, CV_G_, and the effect of cholesterol lowering drugs (CLDs) on lipid levels, and estimated changes per year of biomarkers.

**Results:**

With exception of HDL-C, the long term CV_I_ compared with CV_G_ were higher for serum levels of TC, TGs, and LDL-C in both HIV-1 infected and uninfected men not taking CLDs. Excluding results of TGs in HIV positive men, the CV_I_ compared with CV_G_ were lower for serum levels of TC, HDL-C, and LDL-C in both groups not taking CLDs.

There were significant (p < 0.05) differences in the median serum values of lipid biomarkers among 77 HIV negative men taking and 63 not taking CLDs. Also, with exception of HDL, there were significant (p < 0.05) differences in the median values of TC, TGs and LDL-C among 28 HIV positive men taking or not taking CLDs.

**Conclusion:**

Long term CV_I_ and CV_G_ of biomarkers will be useful for monitoring antiviral therapy side effects on lipid profiles in HIV-infected men. CV_I_ of HIV-infected men for TC, TGs, HDL, LDL were higher significantly than CV_I_ of HIV-uninfected men. Interestingly the long term CV_I_ were higher than CV_G_ for the men, who were on CLDs compared to men not on CLDs. The long-term pattern of CV_I_ and CV_G_ of lipid markers in both HIV-infected and uninfected men on CLDs differed from their short-term pattern.

## Background

Human immunodeficiency virus type 1 (HIV-1) infection, chronic inflammation and treatment with highly active antiretroviral therapy (HAART) has been associated with changes in lipid metabolism, and profiles of blood levels of total cholesterol, triglycerides, HDL-C and LDL-C [1–3]. These changes in the profile of blood levels of lipids is one of the risk factors for cardiovascular and cerebrovascular diseases in HIV-1 infected individuals [4, 5].

An array of sources including pre-analytical, analytical and post-analytical factors affect the outcome of measurement of laboratory biomarkers. To the extent possible, researchers should better understand and eliminate all sources of potential measurement error that can lead to biased findings so that the true biological variations can be detected.

Blood analytes can vary throughout the lifespan of each individual. Some of the variabilities are expected from biological cycles that occur within individuals, while between individual variations may account for another source of variation [6]. Some of the sources such as patient preparation, blood collection techniques, handling, transporting, centrifugation, and specimen storage are controllable, while other issues such as age, ethnicity, and biological sex are not subject to manipulation [6].

Biological changes for many biomarkers occur throughout the aging lifespan as well as in menopause in women. In addition, certain analytes have diurnal, monthly, or seasonal biological cycles or rhythms. However, cyclic variations will not be a major problem for some biomarkers [6–8].

The interpretation of patients’ blood test results using population reference values assumes that the biological variability of the population reference values is similar to the target population [6, 9, 10].

Certain blood biomarkers show a high degree of biological variation (BV) among healthy and unhealthy individuals as an outcome of broad homeostatic set points, even within a reference population that is regarded as ‘healthy’. This individuality between healthy populations makes it a challenging task for laboratorians to generate a reference interval for a blood analyte that represents a ‘healthy’ reference range for all individuals of a given community. Meanwhile, several individuals may have laboratory test values that are very unique and normal for them but still lie outside (5%) or inside ranges of population-based references [11–13].

Repeated quantification of a biomarker obtained by a longitudinal study of healthy and unhealthy subjects may be more desirable to use for evaluation of laboratory results of patients than a single measurement of an analyte associated with population-based references. In the case of repeat measurements of an analyte, the individuals will have their own baseline for the analyte and any changes in value from their baseline result may be associated with the patient’s illness and prognosis [14].

Knowledge of BV of intra-individual coefficients of variation (CV_I_) and inter-individual coefficients of variation (CV_G_) is not only essential for the longitudinal evaluation of blood biomarkers, but it is also important for calculation of desirable quality specification such as assay precision, bias, and total error of a blood biomarker [6].

This study assesses the long-term effect of HAART and cholesterol lowering drugs (CLDs) on biological coefficients of variation, mean values, and individual aging on the basic blood lipid panel of total cholesterol, triglycerides, HDL-C, and LDL-C in HIV-infected and uninfected white Caucasian men over a period of seventeen years.

In addition, we hypothesize that the long-term biological variation of blood lipids in HIV-1 infected and uninfected men on CLDs differ from that of individuals not on CLDs as well as from short term observations.

We accomplished the assessment of short- and long-term BVs by examining the biological coefficient of variation within each individual (CV_I_) and variation between individuals (CV_G_) of the lipid biomarkers in a population of HIV-infected men on HAART and HIV-uninfected men, both groups on or not on cholesterol lowering drugs.

## Material and Methods

### Study Participants

We investigated routine blood levels of lipid panels in HIV-infected and HIV-uninfected white Caucasian men excluding participants with cancers, viral hepatitis B or C, cytomegalovirus (CMV) infections, diabetes or kidney disease. In addition, for the minimization of inter-racial variability, other ethnicities were excluded from this study.

The HIV-uninfected group consisted of 140 white Caucasian men with age ranges between 38–66 and a mean of 51 years old at the start of the study period, all of whom were documented to be HIV-1 sero-uninfected at every study visit (six-month intervals) over the course of study. Seventy-seven out of 140 men were on cholesterol lowering drugs (statins) such as Atorvastatin, Fluvastatin, Simvastatin, Rosuvastatin, Lovastatin, Pitavastatin (*n* = 53) or a combination of statins with the other CLDs drugs such as Gemfibrozil, Fenofibrate, Ezetimibe, or Niacin (*n *= 18), and fish oil, and herbal preparations (*n* = 6).

The HIV-infected group included 90 white Caucasian men 37–64 years old with a mean of 48 years old who were receiving HAART. Sixty-two out of 90 men at the start of the study were on CLDs such as statins (*n* = 45) or combination of statins with other CLDs drugs such as Gemfibrozil, Fenofibrate, Ezetimibe or Niacin CLDs (*n* = 17) during this longitudinal study.

All these individuals were men who have sex with men (MSM) participating in the Los Angeles (LA) center of the Multicenter AIDS Cohort Study (MACS) who self-reported as white Caucasian [15]. The average length of follow-up between a participant’s first and last visit was 17 years.

The institutional review board (IRB) for human studies at UCLA (University of California, Los Angeles) approved the protocols.

### Blood collection and laboratory assays

After informed consent, an overnight fasting blood sample was collected, into one 8 ml SST BD vacutainer tube which was obtained every six months, between 8:00 am to 12:00 pm. Serum was separated within 2- 4 h of the draw and frozen at -80 °C for batch testing. Frozen serum samples for analysis of LIPID panel were shipped to the Heinz Chemistry and Nutrition Laboratory, University Pittsburgh (Pittsburgh, PA). The lipid panel (TC, TGs, HDL-C and LDL-C) were analyzed by chemistry Analyzer AU series (Beckman Coulter, CA, USA) based on the package insert. The methods and assays precision were as follows:

Total cholesterol was measured enzymatically and the intra-assay precision (*N* = 100) and total run were 0.5% and 0.9% for within run and 1.1% and 1.1% CV for total runs (less than 3% CV) for two samples with mean of 115.5 mg/dL and 252.2 mg/dL respectively. Assay sensitivity was 1 mg/dL. Adult reference ranged from 125–200 mg/dL.

Triglycerides was measured enzymatically and the intra-assay precision (*N *= 80) and total run were 0.64%, 0.49%, and 0.51% for within run and 1.65%, 1.41%, and 1.46% CV for total runs (less than 3% CV) for three samples with a mean of 89.4 mg/dL, 191 mg/dL, and 442 mg/dL respectively. The lower detection limit was 0.31 mg/dL. Reference ranges were less than 150 mg/dL.

HDL-Cholesterol was measured enzymatically and the intra-assay precision and total (*N *= 80) were 0.43%, 0.59%, and 0.60% for within run and 2.64%, 2.19%, and 2.12% CV for total runs (less than 3% CV) for three samples with mean of 38.85 mg/dL, 66.39 mg/dL, and 86.33 mg/dL respectively. The lower limit of detection was 1 mg/dL with a reference limit of 40 mg/dL.

LDL-Cholesterol was calculated by the Friedewald equation. The equation is valid if the Triglycerides level is less than 400 mg/dL. The formula is: “LDL = TC- (HDL + (Triglyceride/5)”.

In the equation above TC, LDL, and HDL represent total cholesterol, LDL-C and HDL-C respectively [16]. In cases when the triglycerides level was above 400 mg/dL, the LDL was directly measured. The reference limit was130 mg/dL.

### Statistical analysis

Descriptive statistics of the serum markers that comprised the cholesterol, Triglycerides, HDL-C, and LDL-C, are summarized as means and absolute ranges for both intra- and inter-individual sets of values. Linear trends were estimated using generalized estimating equations (GEE). GEE may be conceptualized as nonparametric extensions to the generalized linear model for longitudinal data [17]. Correlations among the multiple visits from each subject are estimated from data without specifying the exact form of the correlation structure. For every one-unit increase in a covariate across the population, GEE tells us how much the average response would change.

The nested analysis of variance was used for calculation of coefficients of variation of intra-individual (CV_I_) and inter-individuals (CV_G_). CV_I_ and CV_G_ were calculated according to the approach used by Harris and Boyd [18]. All analytes were individually tested using highly precise automated analyzers by the clinical reference laboratories.

The Index of Individuality (II) is the simple ratio of the two biological components of variation: intra-individual to inter-individuals and is calculated using the formula CV_I_/CV_G_ [7]. The II, as defined by Harris [19] assesses the usefulness of population-based reference values for interpretation of laboratory tests. If the II of a given analyte is greater than 1.4, then population-based intervals are useful whereas an II below 1.4 indicates decreased utility of population-based reference intervals. Analytes with an II less than 0.6 demonstrate (paradoxically) a high degree of individuality, making individual-based reference intervals more useful [19]. The Mann–Whitney Rank sum test was used for comparing results between HIV-uninfected and HIV- infected men and closely aged matched to men on statins and not on statins.

Data analyses were performed using SAS version 9.4 (SAS Institute, Cary, NC). Graphs were created using SigmaPlot software version 14 (Jandel Scientific, San Rafael, CA 2018).

Statistical significance was set at the alpha = 0.05 level.

## Results

### Components of biological variation of blood lipid biomarkers

The CV_I_, CV_G_, and index of individuality (II), along with the overall means of TC, TGs, HDL-C, and LDL-C for short term of 1 year (3 visits) and long term of 17 years (34 visits) during follow up are presented for HIV-uninfected and HIV-infected men taking or not taking lowering cholesterol drugs (CLDs) in Table 1.

**Table 1:** Mean values, Intra-individual (CV_I_) and Inter-individuals (CV_G_) coefficient of variation, and index of individuality (II) of blood level of basic Lipid panel of HIV-1 infected and uninfected men

Participants	HIV-1 uninfected Men				HIV-1 infected Men			
Subject number	77 men on CLDs*		**^63 men not on CLDs**		**62 men on CLDs**		**^28 men not on CLDs**	
Visit number	3	34	3	34	3	34	3	34
Follow up year	1	17	1	17	1	17	1	17
Assay	Blood level of total cholesterol				Blood level total cholesterol			
Obs ^#^	215	2255	148	1714	162	1743	66	736
Mean (mg/dL)	196.1	189.9	181.9	193.4	189.4	184.2	165.2	175.1
Standard Error	3.44	3.24	3.91	3.37	5.03	3.63	7.82	6.38
CV_I_ (%)	10.6	16.1	10.2	11.0	13.6	18.6	10.0	13.4
CV_G_ (%)	13.8	14.3	14.7	13.3	18.5	14.9	22.6	18.6
II	0.77	**1.12**	0.69	0.83	0.74	**1.25**	0.44	0.72
Assay	Blood level of Triglycerides				Blood level of Triglycerides			
Obs ^#^	201	2147	132	1577	155	1641	64	638
Mean (mg/dL)	126.8	120.6	91.3	93.1	239.6	188.1	130.2	129.3
Standard Error	8.43	5.32	5.97	4.39	22.25	12.7	19.50	11.54
CV_I_ (%)	40.7	43.8	33.6	36.6	60.4	69.0	40.0	47.0
CV_G_ (%)	50.8	36.6	41.7	34.9	59.3	50.4	90.0	43.7
II	0.80	**1.20**	0.81	1.05	1.02	**1.37**	0.44	1.08
Assay	Blood level of HDL-C				Blood level of HDL-C			
Obs ^#^	215	2252	148	1713	161	1736	67	730
Mean (mg/dL)	47.4	50.8	49.4	55.2	39.6	45.1	45.1	47.2
Standard Error	1.15	1.15	1.11	1.25	1.27	1.41	4.19	3.17
CV_I_ (%)	13.4	15.9	14.1	13.8	16.0	19.5	16.7	19.6
CV_G_ (%)	19.3	19.3	14.2	17.6	22.4	23.9	44.9	34.3
II	0.69	0.82	0.99	0.79	0.71	0.82	0.37	0.57
Assay	Blood level of LDL-C				Blood level of LDL-C			
Obs ^#^	209	2205	147	1696	132	1512	60	654
Mean (mg/dL)	124.1	114.3	113.8	118.6	114.5	105.8	97.8	102.3
Standard Error	3.01	2.91	3.45	3.17	5.40	3.31	5.61	4.28
CV_I_ (%)	15.8	24.9	15.8	15.8	21.4	27.3	16.2	20.8
CV_G_ (%)	18.6	21.4	20.3	20.3	30.9	23.1	25.2	20.6
II	0.85	1.16	0.78	0.78	0.69	1.18	0.64	1.01

^*^ *CLDs* Cholesterol lowering drugs (statins or other), data of ^63 out of 140 HIV-1 uninfected men and ^28 out 90 HIV-1 infected men not on CLDs, Obs^#^; number of available observation data used for the analysis of one and seventeen years follow up

As described in the methods, it is considered appropriate to use population-based reference ranges when the Index of Individuality (II) of a given analyte is greater than 1.4. The indices of individuality for both HIV-uninfected and HIV-infected men in our study for the all markers for 1 year and 17 years follow up were less than 1.4.

The short-term percentage of CV_I_ of TC, TGs, HDL-C and LDL-C were lower than CV_G_ for participants on or not on CLDs in both HIV-infected and -uninfected participants.

The long-term percentage of CV_I_ of TC, TGs, and LDL-C with exception of HDL-C (lower) were higher than CV_G_ for participants on CLDs in HIV-infected and -uninfected participants.

The long-term percentage of CV_I_ of TC, TGs, HDL-C and LDL-C were lower than CV_G_ for HIV-uninfected participants, and the CV_I_ of TC, and HDL-C were lower and TGs and LDL-C were higher than CV_G_ for HIV-infected participants not on CLDs.

In addition, regardless of CLDs, CV_I_ of HIV-infected men for TC, TGs, HDL-C, LDL-C were significantly higher than CV_I_ of HIV-uninfected men (*p* < 0.05).

### Mean and minimum–maximum blood level of lipid biomarkers

**Fig. 1:**
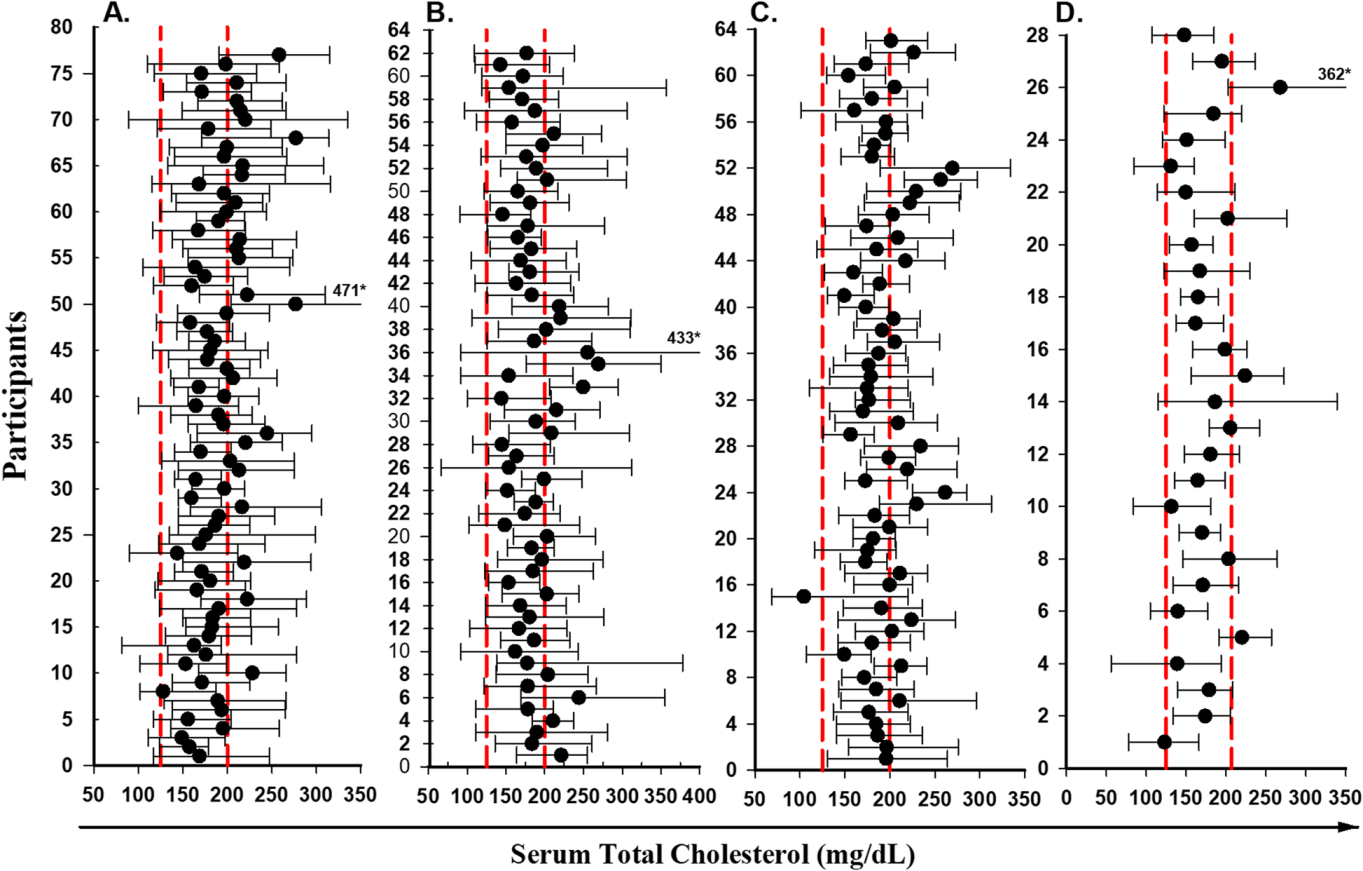
Mean values (black dot) and absolute ranges (error bars) for total cholesterol for HIV-uninfected participants who were on CLDs(A) or not on CLDs(B), vs. HIV-infected participants on CLDs(C), or not on CLDs(D). *absolute maximum value of marker filled outside range of X axis

**Fig. 2:**
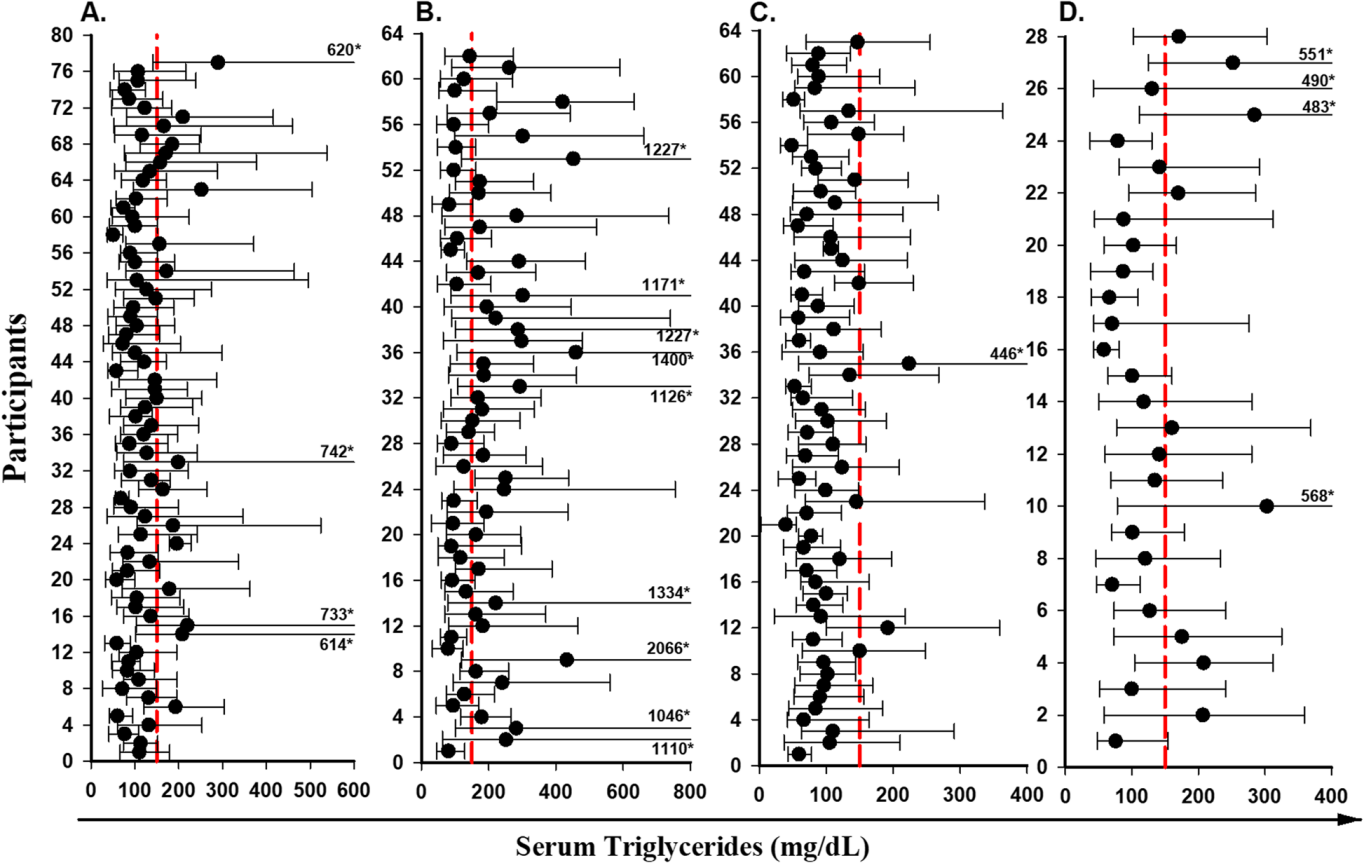
Mean values (black dot) and absolute ranges (error bar) for triglycerides for HIV-uninfected participants who were on CLDs(A) or not on CLDs(B), vs. HIV-infected participants on CLDs(C), or not on CLDs(D). *absolute maximum value of marker filled outside range of X axis

**Fig. 3:**
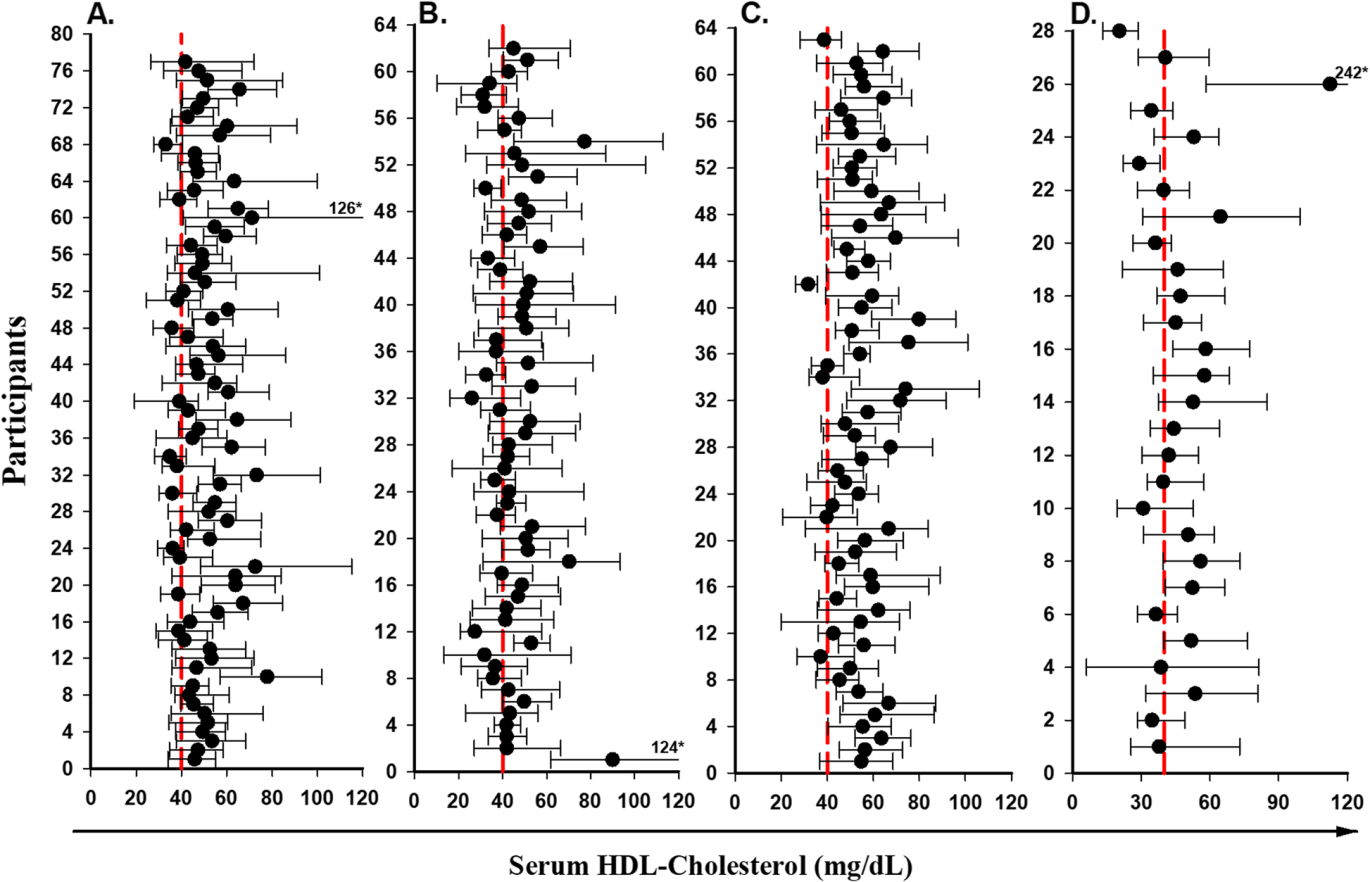
Mean values (black dot) and absolute ranges (error bar) for HDL-cholesterol for HIV-uninfected participants who were on CLDs(A) or not on CLDs(B), vs. HIV-infected participants on CLDs(C), or not on CLDs(D). *absolute maximum value of marker filled outside range of X axis

**Fig. 4:**
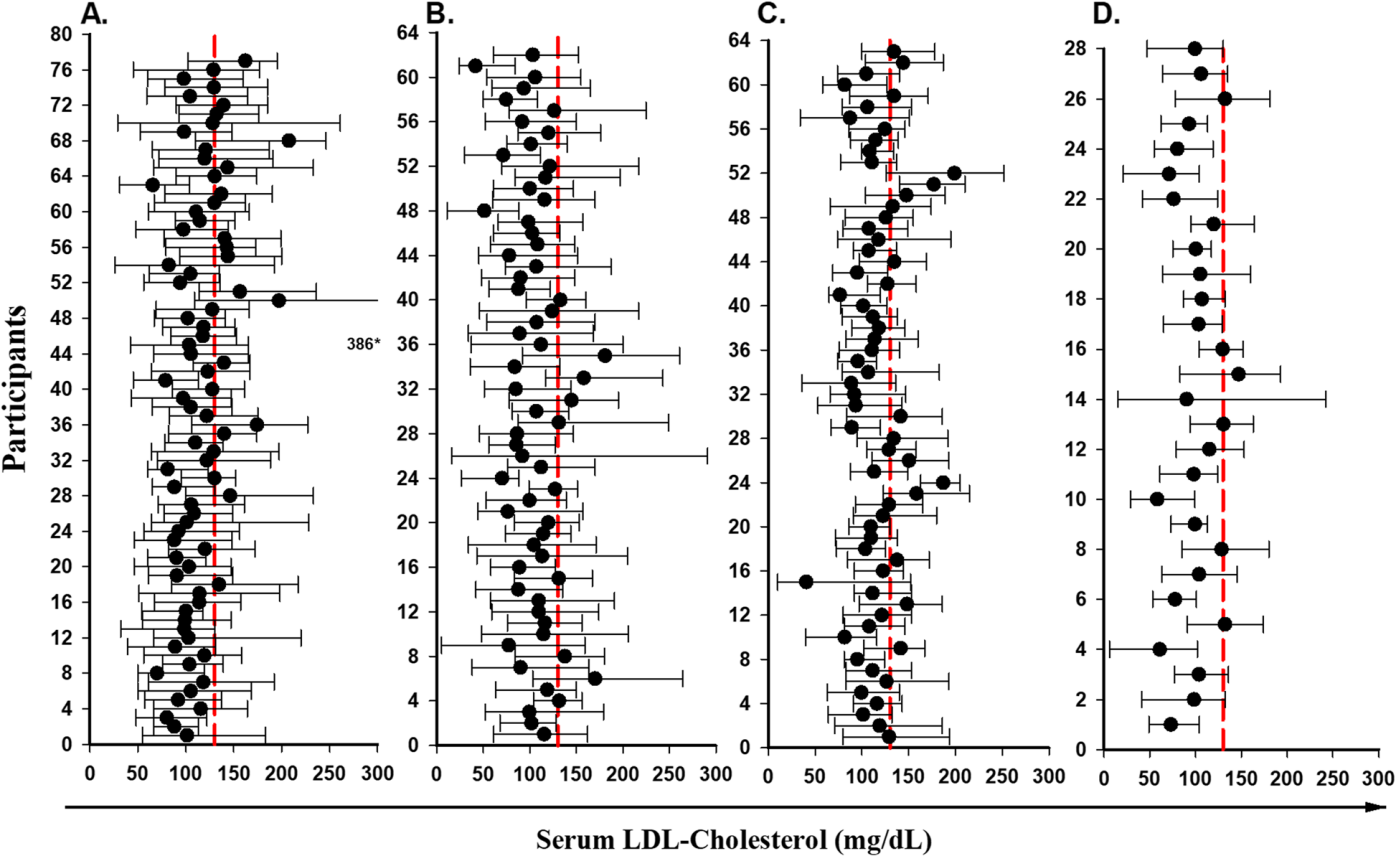
Mean values (black dot) and absolute ranges (error bar) for LDL-cholesterol for HIV-uninfected participants who were on CLDs(A) or not on CLDs(B), vs. HIV-infected participants on CLDs(C), or not on CLDs(D). *absolute maximum value of marker filled outside range of X axis

The long-term mean levels of serum total cholesterol, triglycerides, HDL-C, LDL-C (A-D) and their ranges (minimum–maximum values) over 34 visits for HIV-1 uninfected men with CLDs (*n* = 77) without CLDs (*n* = 63) and HIV-1 infected men with CLDs (*n* = 62) without CLDs (*n* = 28) are shown graphically for total cholesterol in Fig. 1 A-D**,** triglycerides in Fig. 2 A-D, HDL-C in Fig. 3 A-D and LDL-C in Fig. 4 A-D**,** respectively.

Visual inspection of those figures shows that the mean values of the same analyte can differ greatly between individuals in both HIV-uninfected and HIV-infected groups.

The mean serum levels of total cholesterol for HIV-uninfected men were 189.9, and 193.4 mg/dL and for HIV-infected men were 184.2 and 175.1 mg/dL with and without CLDs (Fig. 1 A, C and Fig. 1, B, D) respectively. The majority of mean results of individual men for both groups were within the reference ranges (125–200 mg/dL).

Similarly, the mean, minimum and maximum of serum triglycerides levels for HIV- uninfected men with or without CLDs were 120.6 and 93.1 and for HIV-infected men with or without CLDs were 188.1 and 129.3 mg/dL respectively. The majority of mean results for both groups were lower than the reference limit of < 150 mg/dL (Fig. 2, A, C and Fig. 2, B, D).

The mean, minimum and maximum of serum HDL-C levels for HIV-uninfected men with or without CLDs were 50.8 and 55.2 mg/dL and for HIV-infected men with or without CLDs were 45.1 and 47.2 mg/dL respectively. The majority of mean results for both groups were higher than the reference limit of 40 mg/dL but mean results for 17 HIV-infected men were lower than the reference limit of 40 mg/dL compared to HIV-uninfected men. The lower level of HDL-C in HIV-infected men may be due to HIV infection or HAART. (Fig. 3 A, C, and Fig. 3, B, D).

The mean, minimum and maximum of serum LDL-C levels for HIV-uninfected men were 114.3, and 118.6 mg/dL and for HIV-infected men were 105.8 and 102.3 mg/dL respectively. The majority of mean results for both HIV-infected and HIV-uninfected men were lower than the reference limit of 130 mg/dL (Fig. 4, A, C, and Fig. 4, B, D).

### Magnitude of blood lipid biomarkers changes per year

In order to evaluate direction and magnitude of changes in each marker from baseline over the course of seventeen years of follow up of HIV- uninfected and HIV-infected men who were not on CLDs, generalized estimating equations in SAS software were used to estimate the average change per year in values and corresponding p-values. The longitudinal analysis over the course of seventeen years demonstrated a statistically significant (p < 0.05) ***increase per year*** for blood levels of HDL-C and a decrease per year for TGs of HIV-uninfected men shown in Table 2.

**Table 2:** Average change per year in lipid panels of men not on CLDs over 17 years of follow-up

*Markers*	***HIV-uninfected men (n* = *63)***		***HIV-infected men (n* = *28)***	
	Change*	**Significance**	**Change**	**significance**
Cholesterol (mg/dL)	+ 0.3833 ↔	*p* = 0.109	+ 0.4582 ↔	*p* = 0.250
Triglycerides (mg/dL)	**-0.7658↓**	***p* = 0.05**	-1.8667 ↔	*p* = 0.107
HDL-C (mg/dL)	** + 0.7236↑**	***p* < 0.001**	+ 0.2599 ↔	*p* = 0.152
LDL-C (mg/dL)	-0.1995 ↔	*p* = 0.323	+ 0.1923 ↔	*p* = 0.498

^*^ population average changes in marker values per year; ↑increase; ↓decrease; ↔ no significant change

### Comparison of blood levels of lipid biomarkers of men on and not on CLDs

The Whitney Rank sum test was used for comparing results of blood levels of lipid biomarkers between HIV-uninfected(*n* = 28) and HIV- infected men (*n* = 28). There were significant (*p* =  < 0.05) differences in the median values of total cholesterol (190, 173 mg/dL), triglycerides (82, 104 mg/dL) HDL-C (53.5, 43.8 mg/dL), and LDL-C (116, 102 mg/dL) in HIV-uninfected and infected respectively.

In addition, when we compared the results of TC, TGs. HDL-C, and LDL-C of HIV-uninfected men not on (*n* = 63) and men on (*n* = 63) CLDs we found a statistically significant (*p* =  < 0.05) difference in the median results of TC (190, 184 mg/dl), TGs (81, 100 mg/dL), HDL-C (53.9, 49.0 mg/dL) and LDL-C (116, 110 mg/dL) respectively. For comparison analysis, data were selected based on the age of the two groups. The median results were higher for TC, HDL-C, and LDL-C for men not on CLDs and lower for result of TGs in men without CLDs.

The median value of total cholesterol, triglycerides, HDL-C, and LDL-C of 28 HIV-infected men not on CLDs were 173 mg/dL, 104 mg/dL, 43.8 mg/dL and 102 mg/dL respectively and were statistically significantly (*p* < 0.05) lower for total cholesterol = 184 mg/dL, triglycerides = 133 mg/dL, and LDL-C = 107 mg/dL, and not significantly different for HDL-C = 43.9 mg/dL compared with 28 HIV-infected men on CLDs.

## Discussion

There have been many published studies on short-term biological variation of serum TC, TGs, HDL-C, and LDL-C [20–23]. Short- and long-term longitudinal testing of blood levels of lipids can assist physicians in distinguishing changes in the blood levels of biomarkers over time and during the natural course of an illness in prevention, prognosis, and treatment decisions making.

We longitudinally examined the mean values, CV_I_, CV_G_, and II of serum levels of TC, TGs, HDL-C, and LDL-C of HIV-infected and -uninfected men who were or were not on CLDs every six months for 1 year and 17 years (Table 1).

The short-term mean values were higher for levels of TC, TGs and LDL-C and lower for HDL-C compared with the long-term mean values for both HIV-uninfected and infected men on CLDs. Therefore, the decreases in the mean values of TC, TGs, and LDL-C and increase of HDL-C would be the beneficial consequence of the long-term use of CLDs on blood levels of lipids.

Conversely the short-term mean values were lower for TC, TGs, HDL-C and LDL-C (except for HIV infected participants) compared with the long-term mean values for both HIV-uninfected and infected men not on CLDs. This increase could be due to an ageing effect in individuals on the lipid panel in our study.

The mean values TC, TGs, HDL-C and LDL-C of our study (on or not on CLDs) differed from published studies [24, 25] The selection of white Caucasian men taking or not taking CLDs could account for the differences from other studies.

Knowing intra- and inter-individual coefficients of variation of lipid biomarkers can help the investigator to explore the usefulness of conventional population-based reference values, calculation of reference change value (RCV), and the desirable quality specifications such as assay precision, bias, and total error of blood biomarkers.

The reference intervals will be more useful when the index of individuality (II) is higher than 1.4. A low or high index of individuality of analyte means that the analytes have noticeable or slight individuality respectively [6, 26]. When II is less than 0.6, reference values are limited in detection of unusual results. In contrast when II is more than 1.4 [26]. The index individuality (II) in our lipid markers study with exception of 1 year of HIV infected men not on CLDs were between 0.6 and 1.4. Our data also suggests that an II < 1.4 indicates population-based reference values will have very little diagnostic value, while those may still be a useful tool for patient monitoring purposes. We should always be careful when using population-based reference values to interpret patients’ test results.

The CV_I_ compared to CV_G_ for 1 year of follow up of HIV-infected and uninfected men not on CLDs (excluding TGs) were lower (Table1) and consistent with published short-term duration of 4 weeks; CV_I_ and CV_G_ of the published study was for TC (8.9%, 15.2%), and for TGs (16.8%, 21.1%) respectively [20]. CV_I_ and CV_G_ in a Hispanic Community Health Study/Study of Latinos (HCHS/SOL) from four sites (Bronx, Chicago, Miami and San Diego) with two-time blood collection (baseline and a month apart) were for TC (6.8%, 20.0%), TGs (18.7%, 66.8%), HDL-C (6.4%, 25.7%) and LDL-C (9.7%, 27.4%) respectively [27].

CV_I_ and CV_G_ of the HCHS/SOL study follow the same pattern of CV_I_ < CV_G_ from our short-term observation of men on CLDs and for short and long-term men not on CLDs of HIV-uninfected. Similar CV_I_ < CV_G_ but slight differences in the value of CV_I_ and CV_G_ data to our study which might be this due to differences in the subject population, number of blood draws, study duration, and number of study sites (site to site variability).

In our study the value of CV_I_ and CV_G_ of the blood lipid of HIV-infected men were higher compared with CV_I_ and CV_G_ of HIV-uninfected men not on CLDs. This higher value could be due to the long duration of treatment with HAART medications and their metabolic side effects, higher incidence of opportunistic infections, diet, and/or fat redistribution [4, 24, 25].

There was a significant difference between median values of TC (190 > 173), TGs (82 < 104), HDL-C (53.5 > 43.8) and LDL-C (116 > 102) for HIV-uninfected compared to HIV-infected men not on CLDs respectively. The same observation for HIV-infected participants on HAART of a decrease or little change the serum levels of HDL-C and increase the serum level of TC, LDL-C and TGs has previously been reported [4, 25, 28].

In addition, the median values of intra-individual coefficient of variation (CV_I_) of blood markers (excluding LDL-C) of HIV-infected men were significantly higher compared to HIV-uninfected men.

The median CV_I_ of blood markers in healthy and unhealthy individuals (different illnesses) of HDL-C, LDL-C, cholesterol and triglycerides of published studies (Table 3) were lower compared with the mean values of CV_I_ of our 1, and 14 year follow up study of HIV- infected and HIV-uninfected men not on CLDs [29].

**Table 3:** Median CV_I_ (%) of healthy and chronic diseases in the Ricos C. et al. study and mean CV_I_ (%) of HIV-uninfected and infected men in our study

**Median CV**_**I**_** (%) of Ricos C. et al. **[28]	**HDL-C**	**LDL-C**	**T. Cholesterol**	**Triglycerides**
Healthy subjects	7.1	8.3	6.0	20.9
Chronic liver Disease	-	-	5.2	-
Chronic renal failure	9.5	-	-	15.4
Coronary heart disease	-	9.5	-	-
Diabetes Mellites Type I	7.7	-	7.2	20.0
Diabetes Mellites Type II	-	-	-	-
Hypertension	8.7	-	7.0	21.8
Impaired renal function	-	-	-	-
Lipid disorders	7.1	7.8	5.0	17.8
Mild Hypercholesterolemia	-	6.6	4.7	19.6
*Our study*				
HIV-uninfected on CLDs				
(1 year)	13.4	15.8	10.6	40.7
(17 year)	15.9	24.9	16.1	43.8
HIV-uninfected not on CLDs				
(1 year)	14.1	15.8	10.2	33.6
(17 year)	13.8	15.8	11.0	36.6
HIV-infected on CLDs				
(1 year)	16.0	21.4	13.6	60.4
(17 year)	19.5	27.3	18.6	69.0
HIV-infected not on CLDs (1 year)				
(17 year)	16.7	16.2	10.0	40.0
	19.6	20.8	13.4	47.0

The CV_I_ data in the Table 3 is lower compared with this study. The differences in CV_I_ values from short term observations may be due to differences in gender, ethnicity, lifestyle, age distribution, socioeconomic factors, investigation duration, frequency of blood draws, medications, and chronic inflammation as reported in HIV-infected individuals.

With the exception of HDL-C the pattern of the results of long-term for CV_I_ and CV_G_ (CV_I_ > CV_G_) of the HIV-infected and uninfected white men who were on CLDs differed from that observed in the published study CV_I_ < CV_G_ [13, 27, 30].This difference probably could be due to the effect of cholesterol lowering drugs on the reduction of blood levels of lipids by numerous cholesterol lowering drugs such as statins, fibrate (lowering TGs drug), bile acid sequestrants, nicotinic acid, and proprotein convertase subtilisin/kexin type 9 (PCSK9) inhibitors (LDL-C lowering drug).

Regardless of HIV status, the reduction of lipids by CLDs could be greater between individuals than within an individual. The decrease blood levels of lipids caused by CLDs are independent of the reduction caused by factors such as diet, exercise, lifestyle, smoking, alcohol consumption and stressors, which differ between individuals.

Our data of 17 years follow up of HIV-infected and uninfected men not on CLDs, shows some similarity such as lower CV_I_ and higher CV_G,_ to the published study [13], but not for HIV-infected and uninfected men on CLDs, which show a higher CV_I_ and lower CV_G_ value. This demonstrates that short-term BV of some biomarkers may not be similar to the pattern of long-term BV.

In addition, the effect of aging on the blood biomarkers and estimated average change in values per year and corresponding p-values were calculated for HIV-infected and uninfected men. There was statistically significant (*p* < 0.05) increase per year for HDL and decrease per year for TGs for HIV-uninfected men not on CLDs and no significant changes for HIV-infected men (Table 2).

The limitation of our study was the absence of female participants, missing data for some follow up visits of participants, and using two different methods used for measurement of serum LDL-C. In addition, 28 HIV-1 infected men were not taking CLDs compared to 63 HIV-1-uninfected men in this study.

The strengths of this study, which originated from the MACS longitudinal study population, are the large sample sizes, single-sex, and single-ethnicity population, which maximizes our ability to evaluate biological variation of 17 years follow up of CV_I_ and CV_G_ for HIV-infected and HIV-uninfected men on and not on CLDs. Thus, applying these observations to non-white female groups may not be appropriate.

## Conclusion

Published studies of blood biomarkers demonstrated that the within individual coefficient variation (CV_I_) was less than the between individual coefficient of variation (CV_G_). In our study, for those individuals not on CLDs, with the exception of triglycerides, the results follow the previously published pattern of CV_I_ < CV_G_. However, for the individuals on CLDs, with the exception of triglycerides, our data emerge in the opposite direction (CV_I_ > CV_G_) to the published studies [13, 20, 30]

We believe that this is the first study which shows that within individual coefficient variation (CV_I_) of individuals on long-term CLDs was greater than between individual coefficient of variation (CV_G_). The CLDs are more likely to effect on between individual biological coefficient of variation (CV_G_) than within individual biological coefficient of variation (CV_I_).

The mean values were significantly lower for the levels of TC, HDL-C and LDL-C and were higher for triglycerides in HIV-infected men compared with HIV-uninfected men.

The median of within individual coefficients of variation (CV_I_) of the lipid panel (LDL-C not significant) was significantly higher in HIV- infected men compared with CV_I_ of HIV-uninfected men.

The important “take home” messages are: 1) Data of short-term biological variation of some biomarkers in healthy and unhealthy men are not similar to the patterns of long-term biological variation. 2) Biological variation (CV_I_ and CV_G_) data for healthy and unhealthy men will not be similar depending on HIV status, HIV-1 infected men had significantly higher biological variation for lipid markers compared with HIV-1 uninfected men which it may be due to HIV- infection and HAART. 4) individuals on CLDs, with exception of triglycerides, experienced different patterns of CV_I_ > CV_G_.

The results of this study will be useful for predicting the results of therapy as well as for providing a guide for intra- and inter-individual coefficient variations, identifying the limitations of conventional population-based reference values, and establishing quality goals for different blood biomarkers in both healthy and diseased men. Similar studies in women, and different ethnicities are recommended.

## Data Availability

The datasets used for analysis for this study will be available from corresponding author on reasonable request.

## Abbreviations

BV: Biological Variation
CLIA: Clinical Laboratory improvement Amendment
CLDs: Cholesterol lowering drugs
CMV: Cytomegalovirus
CV_G_: Biological coefficient variation of inter-individual
CV_I_: Biological coefficient variation of intra-individual
HDL-C: High-density lipoprotein cholesterol
HIV-1: Human immunodeficiency virus type 1
II: Index of individuality
LDL-C: Low-density lipoprotein cholesterol
TC: Total cholesterol
TGs: Triglycerides
